# Bone biopsy in chronic kidney disease: still an option?

**DOI:** 10.1590/2175-8239-JBN-2020-0035

**Published:** 2020-05-18

**Authors:** Tilman B. Drueke

**Affiliations:** 1 INSERM U1018, Team 5, CESP, Paris Saclay University, Paris-Sud Univ, UVSQ, Villejuif, France.

Bone histomorphometry remains the gold standard in the assessment and distinction of the different types of renal osteodystrophy. It reflects th sskeletal part of the mineral and bone disorder of chronic kidney disease (CKD-MBD), which also comprises disturbances of mineral metabolism and soft-tissue calcifications^1^. Unfortunately, bone histomorphometry relies on bone biopsies, an invasive procedure. None of the other presently available diagnostic approaches such as non-invasive imaging techniques or circulating biomarkers allows a similarly precise assessment of bone turnover and mineralization status. This being said, bone histomorphometry provides information only at a given point in time, and repeated bone biopsies are even less well accepted by the patients than a single one. Circulating biomarkers allow longitudinal follow-up of bone turnover, although not of bone mineralization or bone volume. Modern non-invasive imaging techniques enable precise evaluation of both cortical and trabecular bone volume and structure but their usefulness in diagnosing bone turnover remains uncertain^2^^,^ 3. 

Patients clearly prefer non-invasive diagnostic procedures to a bone biopsy. The most important question for the patient with CKD is not what is his or her precise type of renal osteodystrophy, but whether the information provided by a biopsy allows a more adequate treatment and avoids clinical events such as bone fractures, hospitalization, and mortality. 

Carbonara et al. set out to examine this issue using the Brazilian Registry of Bone Biopsy (REBRABO)^4^. The study included 260 patients with CKD stages 3-5D and a follow-up of 12-30 months. They assessed available clinical, laboratory, and bone histomorphometry data and classified the bone biopsy findings as osteitis fibrosa, mixed uremic osteodystrophy, adynamic bone disease, or osteomalacia based on bone turnover, mineralization, and volume (TMV) status. The indications for a bone biopsy were heterogeneous: research protocol in 41%, suspicion of aluminum overload in 31%, persistent bone pain in 13%, unexplained hypercalcemia/hyper phosphatemia in 5.4%, nontraumatic bone fracture in 4.2%, planned bisphosphonate therapy in 3.1%, and projected parathyroidectomy in 2.3% of the patients. The authors further assessed the presence and degree of skeletal aluminum deposits. A bone histomorphometry diagnosis was available in only 67% among the 260 patients included at baseline. Osteitis fibrosa and mixed uremic osteodystrophy were the most prevalent forms, present in 51% and 26% of the patients, respectively. Low trabecular bone volume was diagnosed in 44% of patients. The bone biopsy findings were confronted to serum parathyroid hormone (PTH), with 38% of the patients in the KDIGO recommended range, total alkaline phosphatases as biomarkers of bone turnover, with 62% in the KDIGO range, and serum 25-hydroxy vitamin D for vitamin D status, with 45% in the KDIGO range. Hyperphosphatemia was present in 55% and hypercalcemia in 13% of the patients. 

The authors failed to find a specific association between the histomorphometric TMV classification and symptoms or signs, except for a higher prevalence of bone pain in patients with low compared to those with normal bone trabecular volume, and for a higher prevalence of myalgia in the presence of abnormal bone mineralization. Dialysis vintage was found to be an independent predictor of low trabecular volume. Interestingly, there was a high prevalence of aluminum overload not related to the type of renal osteodystrophy but associated with hemodialysis therapy, previous parathyroidectomy, and female gender.

The results were disappointing concerning possible associations of bone histomorphometry findings with the main outcomes of interest, namely non-traumatic bone fracture, hospitalization, and death. Seven patients had a fracture, which was too low to draw any conclusion as to a possible relation with the type of bone disease. There were 56 hospitalizations during the 12-30-month observation time. Fourteen patients died, with 38% of the deaths due to cardiovascular disease. None of the renal osteodystrophy types was associated with hospitalization or mortality. 

The following questions then arise: is bone biopsy useful in predicting patient outcomes? Should only non-invasive diagnostic tools of renal osteodystrophy be used for treatment and outcome prediction? There is no black or white answer, as usual.

A bone biopsy may be very useful to guide treatment options. The 2017 KDIGO guideline states: “in patients with CKD G3a-G5D, it is reasonable to perform a bone biopsy if knowledge of the type of renal osteodystrophy will impact treatment decisions”^5^. Moreover, the guideline suggests that in those with biochemical abnormalities of CKD-MBD and low BMD and/or fragility fractures, one might consider a bone biopsy^5^. As an example, bisphosphonate therapy is contraindicated in CKD patients with low, but not normal, or high bone turnover^6^. The diagnosis of low bone mineral density or low trabecular number and thickness by other imaging techniques certainly is predictive of bone fracture but does not allow distinction between low and high bone turnover disease. In a recent study, even a combination of serum biomarkers of bone turnover and imaging techniques did not allow the precise diagnosis of turnover type^7^. Moreover, imaging techniques do not provide information on bone quality. They are therefore of limited help for treating and preventing renal osteodystrophy and fractures.

The study by Carbonara et al., although suggestive, does not definitively exclude the potential usefulness of bone histomorphometry for predicting clinically important outcomes in patients with CKD^4^. The heterogeneous bone biopsy indications, relatively short observation time, and small number of events are major limitations, as rightfully pointed out by the authors. The inclusion of different CKD stages is another limitation since CKD stage G3 patients greatly differ in many aspects from CKD stage G5D patients. As an additional difficulty, the relatively high prevalence of aluminum overload in the REBRABO patients may not allow a direct comparison with studies on bone-related outcomes in patients with lower aluminum exposure in other geographic regions. Finally, although non-traumatic fractures are directly related to changes in bone quality and mass, numerous causes, other than bone disease, contribute to hospitalization and mortality.

In conclusion, bone histomorphometry is often useful in the diagnosis, treatment, and prevention of fractures in patients with advanced CKD, as again pointed out in a recent position paper by the European Renal Osteodystrophy Initiative group. Its place in predicting clinically important patient outcomes is, however, uncertain, compared with that of circulating biomarkers and imaging techniques.
